# Local-To-Global Hypotheses for Robust Robot Localization

**DOI:** 10.3389/frobt.2022.887261

**Published:** 2022-07-08

**Authors:** R. W. M. Hendrikx, H. Bruyninckx, J. Elfring, M. J. G. Van De Molengraft

**Affiliations:** ^1^ Control Systems Technology, Mechanical Engineering, Eindhoven University of Technology, Eindhoven, Netherlands; ^2^ Robotics, Automation & Mechatronics, KU Leuven, Mechanical Engineering, Leuven, Belgium; ^3^ Flanders Make, Leuven, Belgium

**Keywords:** localization, AGV, AMR, indoor, lidar, global, multi-hypothesis

## Abstract

Many robust state-of-the-art localization methods rely on pose-space sample sets that are evaluated against individual sensor measurements. While these methods can work effectively, they often provide limited mechanisms to control the amount of hypotheses based on their similarity. Furthermore, they do not explicitly use associations to create or remove these hypotheses. We propose a global localization strategy that allows a mobile robot to localize using explicit symbolic associations with annotated geometric features. The feature measurements are first combined locally to form a consistent local feature map that is accurate in the vicinity of the robot. Based on this local map, an association tree is maintained that pairs local map features with global map features. The leaves of the tree represent distinct hypotheses on the data associations that allow for globally unmapped features appearing in the local map. We propose a registration step to check if an association hypothesis is supported. Our implementation considers a robot equipped with a 2D LiDAR and we compare the proposed method to a particle filter. We show that maintaining a smaller set of data association hypotheses results in better performance and explainability of the robot’s assumptions, as well as allowing more control over hypothesis bookkeeping. We provide experimental evaluations with a physical robot in a real environment using an annotated geometric building model that contains only the static part of the indoor scene. The result shows that our method outperforms a particle filter implementation in most cases by using fewer hypotheses with more descriptive power.

## 1 Introduction

Localization is an essential part of an autonomous mobile robot system. The absence of global position sensors and the presence of map disturbances results in challenges that are specific for indoor scenarios. There is a clear trend of deploying robots in indoor environments where they have to be able to robustly deal with changing environments, such as restaurants (Shimmura et al., 2020), hospitals (Cordis, 2020) or nursing homes (Dautenhahn et al., 2015). This raises the expectation of robotic systems on multiple fronts. First, robots are expected to leverage geometric and semantic information from existing sources, which are already available for many indoor environments, such as semantic building information models. This prior knowledge is sensor-independent and can constitute building geometry (walls, corners, columns, doors) or topological information such as room numbers. Second, robots are expected to not only track their pose in a building, but to re obtain their location quickly after failure or reset, without an operator intervening. In prior work, we explored obtaining maps from industry standard building models which are already used to share building data (Hendrikx et al., 2021). In this work we focus on global localization, proposing a method that deals with the ambiguity and uncertainty in the environment on an association level. We show that exploiting local structure first makes the localization outperform a gridmap-based particle filter, while also making it inherently more symbolic and thereby semantically insightful and configurable.

### 1.1 Requirements and Scope

We focus on indoor localization in common public environments. The following requirements have led to this work:

• An existing global map must be available with high-level features for the sensor that is being used, which are explicitly linked to semantic instances of static indoor features.
• The robot system consists of an odometry sensor and a sensor from which features can be extracted that can be associated with the global map features.
• The robot must be able to recover its global location in the environment by making explicit association assumptions, while being robust against stationary *unmapped objects.*

The first requirement assumes that feature instances (e.g., walls, columns) are linked to sensor representations (e.g. lines, corners) as described in Hendrikx et al. (2021). In this work we use a system equipped with wheel encoder odometry and a conventional 2D planar laser scanning device. We assume that static environment features are represented well by straight or circular geometries on an existing feature map.

### 1.2 Proposed Method

**FIGURE 1:**
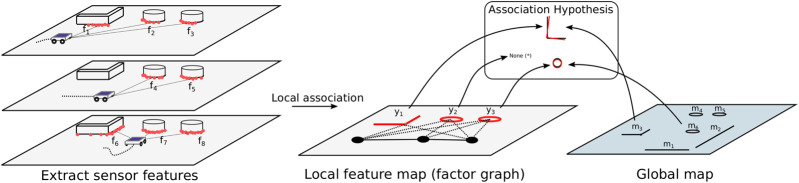
A graphical depiction of the method we propose in this work. A single hypothesis is shown as an example.

Our method localizes globally by first combining local *sensor features* (geometric features such as lines, corners and circles) from separate scans into a consistent *local feature map*. These *local features* are then associated with *global map features* using an association tree. The levels of this tree represent the local features, and the nodes represent the possible association of the local map feature with a global map feature. We evaluate association likelihood based on a moving horizon strategy and prune unlikely associations based on feature description and spatial congruency, leading to a hypothesis tracking approach where the sample space is that of data associations. Note that our method is sensor-independent and well-suited to function with different or multiple sensors modalities. Our methods deals with *unmapped objects* on a feature association basis and does not rely on a map containing all visible geometry. We assume that the features on the global map are geometrically accurate and that objects that have velocity do not resemble our static features. The method is graphically depicted in Figure 1.

## 2 Related Work

Dealing with multiple hypotheses, one can distinguish between location-driven and feature-driven localization approaches as remarked by Arras et al. (2003). Location driven approaches maintain hypotheses using a sample set on a continuous space, or use an *a-priori* selected topological graph in which the nodes represent locations in the environment. On the other hand, feature-driven approaches explicitly maintain hypotheses on associations between features that are seen locally and features on a map. The latter characterizes our method; however, relevant prior work exists using both approaches which we will now consider.

### 2.1 Hypothesis Tracking Methods

A reference work for our method is Arras et al. (2003), in which the authors perform global localization in an environment described by geometric (line and point) models. They maintain a tree of local-to-global data associations together with multiple extended information filters (EIF) to solve for the map location of the robot. They employ both binary constraints to check if feature pairs are metrically consistent both locally and globally and, when possible, use rigidity constraints to check the likelihood of a new pairing given the location based on old pairings. Rather than a Bayesian approach, they use an approach that designates hypotheses as equally valuable options, and use a *lack-of-fit* measure only in their duplicate removal strategy. They also use an explicit clutter hypothesis and verify their approach in an experiment. He and Hirose, (2012) expand on this work by introducing a relation table that captures the coordinate independent local structure of a map of line segments in a more efficient way. They check the correspondence of line segments (congruency under rotation and translation) on a local and a global map by introducing a geometric relation comprising two lengths, an angle and two relative distances between the segments. Using this relation they propose depth-first constraint-based search in an interpretation tree to find matching line segments for localization without a prior pose being available, and account for an *unmapped item* option (*null features*). Their work first applies pair-wise constraints to balance the complexity of the search in the tree and afterwards checks an alignment constraint of the total map based on a least-squares solution. They limit the total number of *null features* allowed in order to mitigate search time, and mention that determining this parameter is environment-dependent. The authors also use a topological representation (e.g., hallway, room) of their environment with annotated visible line segments for each location, in order to restrict relation matches to simultaneously visible pairs. The authors mention Multiple Hypothesis Tracking (MHT) but do not expand on the details of their MHT implementation. They provide a comparison of their method and Monte Carlo Localization (MCL) in a lab environment with clutter objects added and show increased performance. Our method draws inspiration from certain aspects of the two mentioned works, as we will elaborate on later. Other works for different sensor modalities such as (Lutz et al., 2011) provide a camera-feature based multi hypothesis approach that searches for feature descriptor matches in a persistent database and then applies a Kalman update to all possible matches. Matches are Mahalanobis-distance gated and pruned based on the relative number of observed landmarks with respect to other hypotheses. Morris et al. (2014) take an approach using hypotheses on submaps that are scored against the global map based on the innovation they provide to the local odometry trajectory. They use grid based representations and the generation of hypotheses is not within scope of their work. Hong et al. (2017) use a nearest neighbour filter and a computer simulation to show global localization without unique landmarks. However, they assume that they can initialize hypotheses by inverting the measurement function to obtain a unique state which is not always possible. A more recent reference work is (Gao et al., 2019) which focuses on 2D LiDAR global localization using structural unit encoding and multiple hypothesis tracking. The authors mark certain scans from their map as *key scans* and determine endpoint line features. They then form a set of structural units, containing the relations between line endpoints and line angles which is rotation invariant. They then quantize this set using a soft encoding scheme that does not require training on prior data. An exhaustive search is then used to find matching poses from candidate key scans. The authors then use a multi hypothesis tracking approach where they let the matching poses be the priors, and use the odometry as a likelihood measure to prune unlikely hypotheses from the tree. They compare with the well-known Adaptive Monte Carlo Localization (AMCL (Fox, 2003; Thrun et al., 2005)) method and find improved results. However, their method pre-processes the map based on existing scans, while our method does not require existing scan data.

### 2.2 Data Association for Simultaneous Localization and Mapping

Data association methods are important for Simultaneous Localization and Mapping (SLAM), where they are required for reliable loop closures. One method to deal with finding associations is Random sample Consensus (RANSAC) (Fischler and Bolles, 1981), which randomly selects pairings and evaluates whether the resulting model parameters lead to an acceptable amount of inliers. A large disadvantage is, however, the limited control it provides over the search effort and the fact that it is not guaranteed to find a set of hypotheses in acceptable time. A more structured method is Joint Compatibility Branch and Bound (JCBB) (Neira and Tardós, 2001; Shen et al., 2016). It searches the so-called *interpretation tree* for the best correspondence between a set of features extracted from sensor data and the map. The method is more reliable than nearest-neighbor association or pair-wise consistency evaluation, as these methods do not take the correlation into account between different observations from the same robot pose. JCBB is shown to be more precise because it evaluates the compatibility of all features seen from a current pose. While the tree structured search of JCBB is similar to our approach, JCBB is not a *hypothesis tracking* approach and as such does not evaluate compatibility over a horizon of motion while maintaining multiple viable pairings. This makes it unsuitable for global localization where different locations appear similar. The use of hypotheses over data associations has also been researched extensively in the context of graph optimization frameworks, see e.g., (Latif et al., 2014; Cadena et al., 2016a,b). In early works such as (Thrun and Montemerlo, 2006) the authors discuss for a large scale SLAM approach how earlier data associations can be evaluated based on the error they induce on later data associations. They provide a greedy correspondence test to deal with the correspondence problem, but note that other methods may be favourable. Other works such as Davey, (2007) and Olson and Agarwal, (2013) focus on methods that either use expectation maximization methods to iteratively search for the data associations or they focus on back-end heuristic methods such as switching variables and robust error models, which allow recovery from false associations. These methods do however generally not provide the multi-hypothesis output on a semantic association level or are only applicable to tracking with a good initialization. Another important work in robust localization that also focuses on existing floor plans is (Boniardi et al., 2019), where the authors show how to use these floor plans as priors for localization and maintaining an updated map based on a pose graph framework. However, the authors do not focus on global localization in their work.

### 2.3 Particle Filter Methods

Particle filters deal with the problem of global localization by covering the location state space with a discrete set of samples (hypotheses) generated from a proposal distribution. They are currently a popular method for representing hypotheses in the pose space (see, e.g., Fox et al. (2003); Elfring et al. (2021); Thrun et al. (2005)). The combination of particle filters with LiDAR sensors is often considered a *state-of-the-practice* solution to robot navigation in indoor scenarios. Not in the least because easy-to-use reference implementations exist. A recent work on global localization is (Aycard and Brouard, 2020), where the authors add an initialization step to a particle filter that uses indexes generated on the map to find suitable initial positions for the particle filter. They show that this initialization step greatly reduces the initial number of particles needed to let the robot localize itself. The authors pre-compute a set of laser readings in a grid map for over a million positions and use a two-level index to retrieve initial positions and orientations that are likely to match the sensor reading.

### 2.4 Place Recognition Methods

Some of the methods mentioned such as Aycard and Brouard, (2020) incorporated ideas from information retrieval. These methods are often used with cameras and reference image databases (see e.g., Barros et al. (2021)) and may be used to speed up association initialization but are not exhaustive, and may not work well with existing maps and LiDARs because of lack of unambiguous local context. Furthermore, they often require training on large data sets that are processed to determine a vocabulary, and this is not readily available in every environment. The methods also do typically not include the motion of the robot to gather context or exploit the online recursive nature of the localization process and are therefor significantly different from our proposed method.

### 2.5 Contributions

The contributions in our work to the state of the art are the following:

1. We group measurements into local features of type *line*, *circle* and *corner*, resulting in a sparse and accurate local map that is associated with the global map.
2. We assess the lack-of-fit using a horizon of these features that includes the new candidate association, without resolving the robot pose to a belief distribution.
3. We perform an experimental comparison with a commonly used particle filtering method and discuss the trade offs.

In contrast to the works (Gao et al., 2019; Aycard and Brouard, 2020), which also perform global localization using 2D LiDAR, we do not rely on grid maps or raw sensor data as a prior and as such do not leverage data-driven heuristics. We specifically aim to use object-based prior representations that can be obtained from other sources as well (e.g. Hendrikx et al. (2021)) in the form of vector maps. This makes or work more similar to Boniardi et al. (2017); however, the authors do not focus on global localization in their work. Finally, our work draws inspiration from Arras et al. (2003); He and Hirose, (2012) as we consider similar map representations and features. However, their work hypothesizes on individual measurements and Arras et al. (2003) attaches information filters to individual hypotheses. Furthermore, He and Hirose, (2012) uses only line features and does not elaborate on their hypothesis tracking mechanism. Our approach reduces the data association space by first associating features locally, and is more in line with how humans reason about localization (e.g., we merge local context from different viewpoints into local features before deciding to relate observations to a map). Furthermore, our framework employs a Bayesian strategy to keep track of hypothesis likelihood. Finally, we compare our work to a often used grid map based particle filter, where the grid map only contains static geometry. The rationale behind this comparison is that grid maps can be easily generated from prior maps as well and provide access to the static geometry without relying on features to be detected, which brings an important trade-off to light. In addition to quantification of our method, we also show conceptual advantages in the form of increased insight in the associations of the method when compared to traditional approaches. Our implementation is available in our repository.^1^

## 3 Preliminaries

In this section we introduce preliminary concepts and explain the rationale and assumptions that underlie our method. We approach localization from a *data association* point-of view. Our goal is to have correct associations from *local* (i.e., sensed) feature measurements to features on the global map. In general, these associations are dependent on the global pose, and obtaining one from the other is straight forward. We argue that the benefit of data associations is that they form a finite discrete sample space that can indirectly represent pose distributions efficiently that must otherwise be approximated by samples. It is possible to calculate the belief over global pose from associations (or vice versa) from the general probability distribution:

$$\underset{D^{z},M,X^{m}}{\max}P\left(D^{z},M,X^{m}|Z\right).$$ (1)

Where $D^{z}=\left\{D_{1}^{z}\ldots D_{n^{z}}^{z}\right\}$ are the data associations of the *n*_*z*_ individual measurements $Z=\left\{z_{1}\ldots z_{n^{z}}\right\}$ (for instance, bearing measurements) with the *n*_*m*_ features on the *global map* $M=\left\{m_{1}\ldots m_{n^{m}}\right\}$ . This general representation allows to solve over the data associations, robot poses in the map frame $\left(X^{m}=\left\{X_{1}\ldots X_{n_{x}}\right\}\right)$ and map features simultaneously (assuming that the global map is probabilistic). Evaluating (1) over both the continuous variables and the association space $D_{1}^{z}\times \cdots \times D_{n^{z}}^{z}$ is generally unnecessary because of conditional independencies. In our method, we first reduce the data association space by forming a local feature map **Y**, exploiting the fact that measurements can be combined locally into features first and are correlated. Furthermore, we assume that the global map that is available is accurate and that the relative local map coordinates are accurate enough over short horizons of measurements to be independent of the global map, given the measurements. Let **Y** be the local map features (e.g., the coordinates of point landmarks) corresponding to multiple measurements, and **X** be the local robot poses. We assume that the *maximimum a-posteriori* (MAP) estimate:

$$X^{*},Y^{*}=\mathrm{arg max}P\left(X,Y|Z\right),$$ (2)

in a local frame *ℓ*, can be used to estimate the data associations *D* ^*y*^ of the local features with the global map. Here we assume that the data associations in the local map can be reliably made. The resulting space of *D* ^*y*^ is much smaller than $D^{z}$ . The remaining problem then becomes to estimate the data associations $D^{y}$ given the local map estimate:

$$\underset{D^{y},T_{\ell }^{m}}{\max}P\left(D^{y},T_{\ell }^{m}|Y^{*},M\right).$$ (3)

Where the local map estimate horizon is chosen small enough and Y* is recomputed for different evaluated patches, such that the assumption of local metric accuracy is met. The pose $T_{\ell }^{m}$ denotes the registration of the local map with the global map, which may be of interest depending on the type of motion control that is used (i.e., *local feature-based motion control* or *global path following*). Because we focus on global localization, we explicitly choose to solve a local factor graph only, and do not introduce the global priors in the estimate. This results in only a single factor graph having to be solved as opposed to multiple. Furthermore, when *global localization* is the current robot task, motion actions and local sensing can (and should) be performed in such a way as to achieve reliable local estimates (e.g., slow driving and conservative detection policies).

### 3.1 Local State Estimation

The state of the *local feature map* is represented using primitive features which are estimated together with robot poses using a graph-based optimization, as is common in SLAM literature. We denote the robot pose at time *t* as $x_{t}^{\ell }={\left[x_{t},y_{t},\theta_{t}\right]}^{T}$ in a local frame *ℓ*. The pose of this frame is arbitrary (usually the starting location of the robot), and is only used for intermediate representation of numeric coordinates. We represent the state of local landmarks by the variables $y_{i}^{\ell }$ which are measured by feature measurements **z**_*j*_ from the robot poses. If we denote the current optimization horizon of robot poses and landmarks by the sets **X** and **Y**, and the relevant set of measured variables by **Z**, we can write the maximum a-posteriori estimate of the these variables given the measurements as:

$$P\left(X,Y|Z\right)\propto P\left(Z|X,Y\right)P\left(X\right)P\left(Y\right)$$ (4)

Where we have assumed independence of measurements. The measurement likelihood *P* (**Z**|**X**, **Y**) in the above equation is encoded by *factors* in a *factor graph*. These factors take the form of nonlinear measurement functions *h*_*j*_ of the state where the uncertainty is represented by additive zero-mean multivariate Gaussian error, taking the form:

$$P\left(z_{j}|X_{j},Y_{j}\right)=∥h_{j}\left(X_{j},Y_{j}\right)-z_{j}{∥}_{\Sigma_{j}}$$ (5)

Where $∥\cdot {∥}_{\Sigma_{j}}$ denotes the Mahalanobis distance, using the measurement covariance matrix Σ_*j*_. A nonlinear least-squares problem is obtained by taking the log of the product of factors, leading to the formulation:

$$\log P\left(X,Y|Z\right)\propto -\overset{K}{\underset{j=0}{\sum }}e_{j}^{T}\Sigma_{j}^{-1}e_{j}$$ (6)

where we use **e**_*j*_ = **h**_*j*_ (**X**_*j*_, **Y**_*j*_) − **z**_**j**_ for all *K* measurements. The resulting problem is a minimization over the negative log likelihood, resulting in the non-linear least squares formulation:

$$\underset{X,Y}{\min}\overset{K}{\underset{j=0}{\sum }}e_{j}^{T}\Sigma_{j}^{-1}e_{j}$$ (7)

where we assumed uninformative priors, leaving only factors in the optimization. The resulting maximum a-posteriori (MAP) estimate contains the most likely local map and robot poses given the measurements. Here we assume that all data associations between local features and their measurements are correct and can be reliably made. It is often possible to design features and feature detectors with enough saliency and discrimination such that this assumption holds locally, given proprioceptive sensing that is relatively accurate over short distances. We use the GTSAM library to perform the optimization.

### 3.2 Geometric Primitives

**FIGURE 2:**
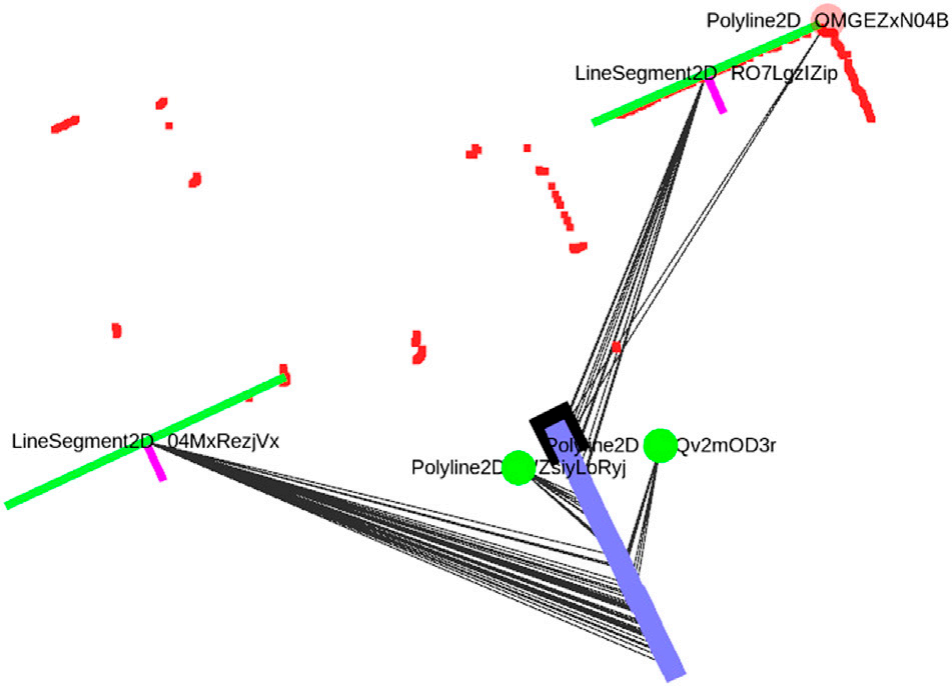
A small locally consistent map of line and corner features, that has been obtained by locally associating and merging feature representations and solving the factor graph optimization problem after each new LiDAR scan is obtained. The robot poses are shown in blue with the current pose in black. measurement associations are shown as grey lines and current LiDAR points in red. The line measurements include a normal to indicate their visible side. A corner detection is shown in translucent red when not enough measurements have been associated with it (candidate detection). The features get assigned unique string identifiers.

We use the term *measurements* to describe the primitive *sensor features* that are extracted from raw sensor data and that are associated with features on the *local map*. We refer to the latter as *local map features* which can be associated with multiple measurements, and their geometry must be consistently inferred from the *sensor features* belonging to the measurements. An example of a local map feature can be a circle, which is associated with multiple circle sensor features from different LiDAR scans. Measurements also contain *spatial* information about the location of the features with respect to the robot. These are in the form of *range/bearing/pose* measurements. In this work we use three *geometric primitives* that form the basis for landmarks in our localization approach: *line segments*, *corners* and *circles*. Note that these features resemble three general geometric constraints on the state of the robot given the measurement, by reducing the likely state to either a line segment, a pose or a circle around a point measurement. These feature types can therefore be generalized to other sensors as well. A line segment is parametrized by two points which represent the *begin* and *end* of the line segment. A *partial* line segment is any combination of two points that are contained within a line segment, as is often the case for LiDAR measurements. A corner is modeled by a *polyline* consisting of three points, i.e., two line segments sharing a common point. Note that a LiDAR allows to detect approximate end-points of lines, by evaluating whether the neighboring range extends beyond the line segment or is visually obscured by something closer. Finally, a circle is modeled by a center point and a radius. An example of a local map created from sensor data can be seen in Figure 2. We will now proceed while assuming that such a local map is available. A more detailed discussion on the mechanisms used to create the local map has been deferred to Appendix A.

## 4 Localization Approach

In this section we will explain our global localization method. We will first elaborate on how we maintain our local map in relation to our global objective and then introduce the global association approach using a hypothesis tree.

### 4.1 Local Mapping

The map that we maintain locally is updated for each new sensor feature. Once a new sensor feature is extracted, the map is updated using the approach described in Algorithm 1.

Algorithm 1 The algorithm used to update the local map features when a new set of sensor features has been extracted from a laser scan.

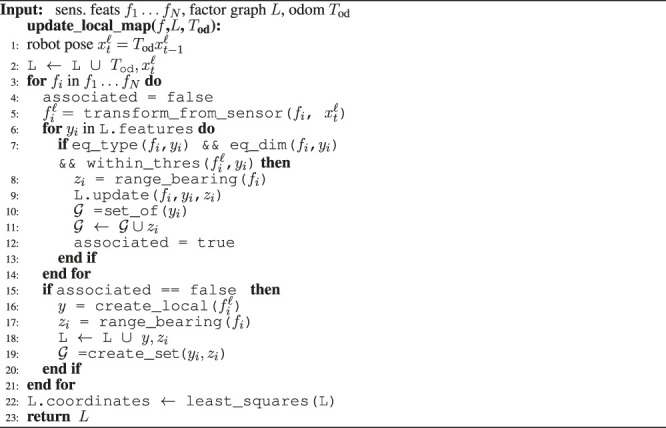

The sensor feature extraction is triggered by a distance monitor based on wheel odometry and the latest available sensor reading is combined with a time-stamp interpolated version of the odometry encoder reading. The updating routine (Algorithm 1) shows how a sensor feature is first checked for existing associations with the local map. Here, the functions eq_type () and eq_dim () check if the feature has the right type and dimension (e.g. a circle with similar radius). The function within_thres () checks if the feature is close enough to be associated, where we use a Euclidean distance. If no such association exists, a new local map feature is instantiated. For this purpose, we introduce the notion of a measurement set $G_{i}$ which contains a local map feature and all measurements associated with it^2^.

### 4.2 Global Association Tree

We maintain hypotheses over the space of data associations between the local and global map. These hypotheses will be maintained based on explicitly configurable assumptions, while a Bayesian approach is used to update scores for hypotheses. Consider a snapshot of a given local map with a feature *y*_*i*_ ∈ **Y**. We define the association variable *D*_*i*_ as follows:

$$D_{i}=\left\{\begin{array}{c} d_{i}^{1},\;\;\;\;y_{i}\mathrm{originates}\left(\mathrm{partly}\right)\mathrm{from}\;\;m_{1}\;\\ \ldots \;\\ d_{i}^{n},\;\;\;\;y_{i}\mathrm{originates}\left(\mathrm{partly}\right)\mathrm{from}\;\;m_{n}\;\\ d_{i}^{*},\;\;\;\;y_{i}\mathrm{originates}\mathrm{from}\;\;\mathrm{unma}\mathrm{pped}\text{object}\; \end{array}\right.$$ (8)

**FIGURE 3:**
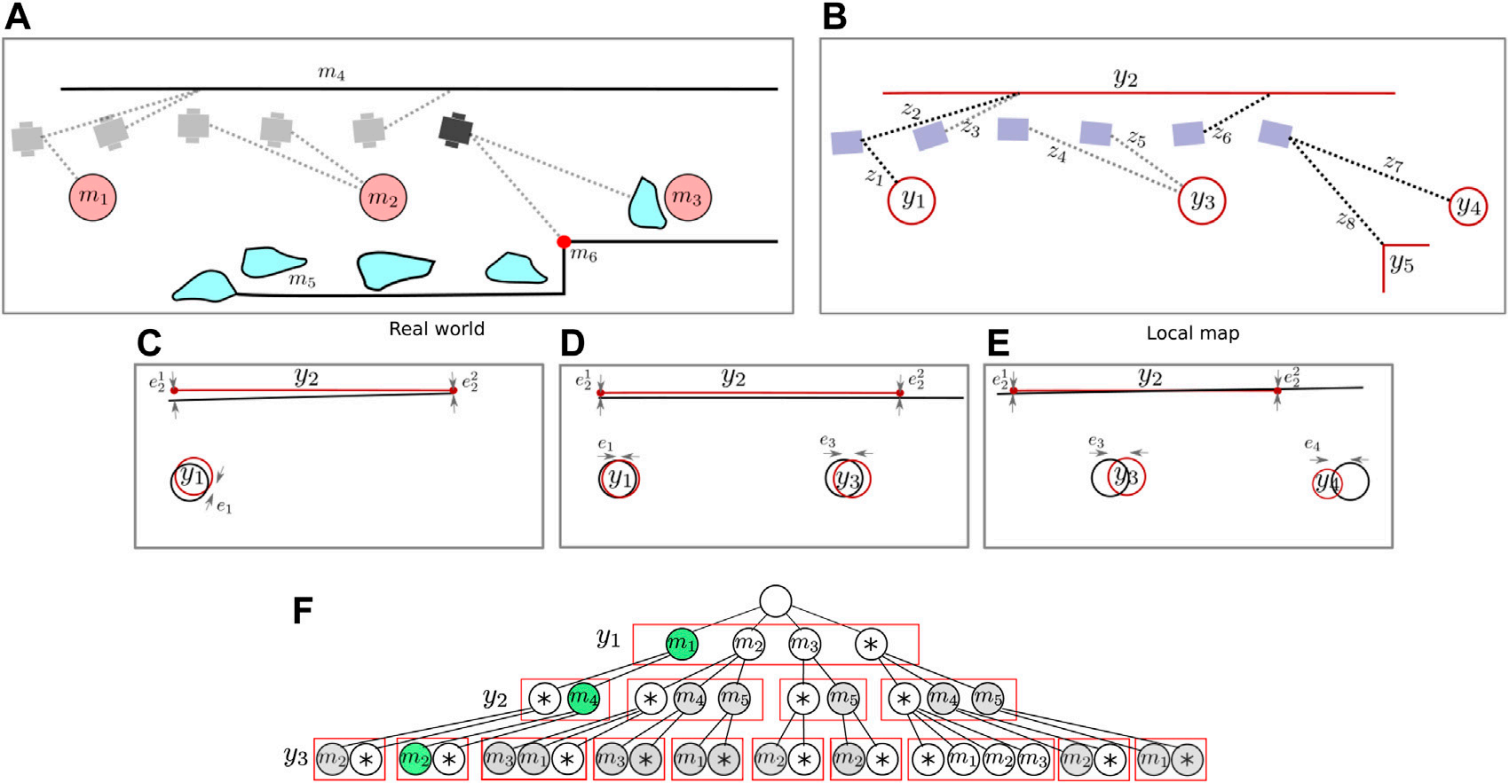
A basic example showing the global association tree. As the robot drives in the real world **(A)**, it builds a local map of features **(B)**. The local measurements are associated with these local features, and added to their set. Nodes are then used to represent an association between a local feature and a feature on the global map in **(F)**. In this example, the robot first spots a circular feature. After spotting it multiple times, the consecutive measurements are added to a set and a local feature is instantiated. Possible pairing hypotheses with the global map are generated based on feature type and dimensions. When a new line segment is detected, a second layer of hypotheses is added to the tree based on spatial consistency between the circle and line feature on the local map and the global map. Finally, an *unmapped object* is spotted which has similar geometry to a column, highlighting the importance of allowing for a *unmapped object* option in the association tree, although these levels are not visualized anymore. In sub figure **(C–E)** the spatial congruency is visualized, which is used together with the feature’s descriptive component to accept or reject association hypotheses.

**FIGURE 4:**
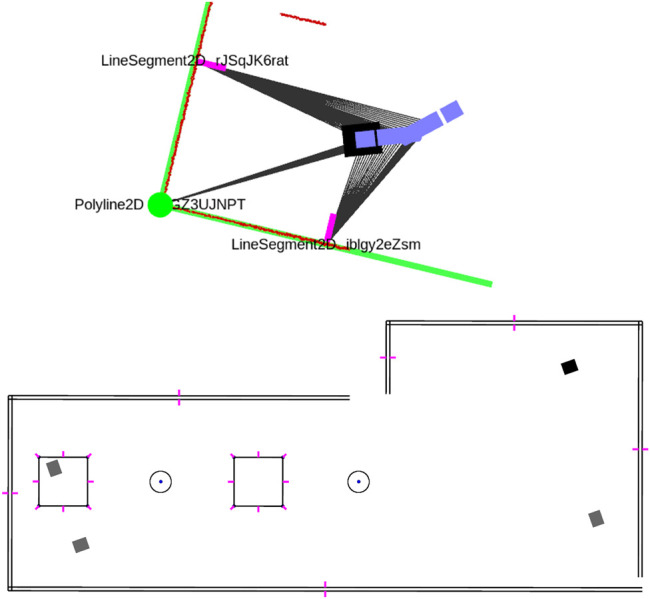
The global map hypotheses (bottom) corresponding to a local map (top). Based on the local features in that figure, four possible map poses are shown (not all hypotheses permit showing a unique pose). Note that walls have been modeled by double line segments with visibility normals visualized by small purple segments. The labels of the global map have been omitted in this example.

Here, *unmapped object* refers to something that is not on the *global* map. We explicitly mention *partly* because we allow cases where the global feature *m*_*j*_ is responsible for only part of the geometry of *y*_*i*_, as long as the effective constraints is the same (e.g. a wall that appears extended by an object from a certain viewpoint). We will now consider how the association variables form the association tree that we will refer to in the remainder of this work. Having a local map available, we evaluate Eq. 3 by starting with the first feature and expanding the tree for each consecutive feature. The *levels* of the association tree coincide with the local map feature indices, i.e., nodes *h*_*ij*_ on level *i* pair *y*_*i*_ with a global map feature. The leaf nodes of the tree represent an association assignment of all local map features up to *y*_*i*_, obtained by following the parents par (*h*_*ij*_) of the nodes upwards. The leaf nodes thereby form the hypotheses under consideration. Each node is thus associated with a local feature and its measurement set, a global feature associated with it and a parent node^3^. Nodes on a single level *i* can associate a local map feature *y*_*i*_ with the same global map features, if their parents are different. We can require a measurement set to meet certain constraints on the measurement set and local features before an association is made or revisited. For example, we can require a certain minimal number of measurements from different poses. The structure that we describe naturally forms a hypothesis tree T and the current *sample space* is formed by the *leaf nodes*, which all represent a unique set of data associations between local map features and global map features. Note that because of the inclusion of *unmapped object* associations, this sample space is *exhaustive* when no pruning has been applied. An example is provided in Figure 3. As the robot drives, it detects a circular feature, and a set $G_{1}$ is created. Based on the shape and size of the feature, a pairing can be made with possible features on the map. Alternatively, it can be something that is not on the map (*). When a next line segment feature is spotted, a new set $G_{2}$ is formed and new leaf hypotheses are formed for each old leaf hypothesis. Based on the old hypothesis, we will now consider when associations are feasible and when they are not. Feature shape is used first to select plausible candidates, after which we assess whether the *spatial structure* for a small horizon of recent features is congruent with the spatial structure obtained from the map (Figure 3C). This amounts to assessing the *spatial likelihood* of the local map (Figure 3B) given the data associations. Depending on the specific representation of the map, features and uncertainty, the likelihood can take many forms (e.g. topological, semantic or spatial). But we choose to do a spatial registration step and assess whether the resulting spatial mismatch is small enough to refrain from rejecting the pairing hypothesis. At this stage, making as little feasible hypotheses as possible and pruning unlikely leaves becomes necessary to avoid a combinatorial explosion. For hypotheses marked in grey, a unique maximum likelihood pose on the map can be determined based on the feature pairings. However, for assessing the geometric *lack-of-fit* of local detections, such a pose is in general not necessary (e.g. in the case of parallel line segments). In Figure 4, a simulation example shows four likely hypotheses on a global map given the detections on a local map.

### 4.3 Hypothesis Likelihood

Having a window of local map features available, the problem under consideration is to determine which association hypotheses are likely and which are not. The main mechanism to keep a small set of hypotheses is to prune unlikely associations immediately from the tree. Scoring hypothesis likelihood recursively is not a strict necessity to achieve this in our method, as we rather use more direct approaches for rejection. However, maintaining likelihood is useful for three reasons: 1) it allows to keep the most likely hypothesis when hypotheses are similar (e.g. they share recent associations), 2) it allows to prune the least likely hypotheses when the number grows too large to maintain efficiently and 3) it would allow a planner to optimize actions taking relative uncertainty into account. Regarding the second remark, we note that our method guarantees a qualitative distinction between hypotheses based on their data association, making rigorous pruning possible while still keeping enough descriptive power in the remaining set.

In general, localization does not reduce to merely a *lack-of-fit* evaluation problem, because the likelihood of the associations we make with local map features (including the *unmapped object* pairing) is also dependent on the *local structure* of the feature (e.g. shape, size, semantics) which is independent of position. Furthermore, the nature of disturbances in the real world and the feature density on the map play a role in determining prior likelihood for a global association. We now explain our Bayesian strategy for maintaining a *breadth-first* expansion of the tree, allowing us to express confidence using models of both spatial and descriptive likelihood. We employ a *gating* or *constraint* procedure based on feature description and spatial error, leaving us with only a certain subset of hypotheses that should be considered likely. In general, we can evaluate the association probabilities using the product rule:

$$P\left(D|Y^{*},M\right)=\overset{n^{y}}{\underset{i=1}{\prod }}P\left(D_{i}|D_{i-1:1},Y^{*},M\right),$$ (9)

followed by Bayes rule to evaluate in terms of spatial likelihood and prior:

$$P\left(D_{i}|D_{1:i-1},Y^{*},M\right)\propto P\left(Y^{*}|D_{1:i},M\right)P\left(D_{i}|D_{1:i-1},M\right).$$ (10)

Where the spatial likelihood allows us to evaluate whether the local map that we observe is coherent with the global map given the data associations. In other words, once we evaluate a new feature pairing *D*_*i*_ for a local map feature *y*_*i*_, we evaluate the spatial likelihood $P\left(Y^{*}|D_{1:i},M\right)$ of the local map, given the feature pairing and its parent pairings up to a horizon *N*_*d*_ resulting in a recursive evaluation. We can also incorporate a prior $P\left(D_{i}|D_{1:i-1},M\right)$ for this pairing, expressing confidence based on feature description (e.g. semantics, shape, size). In practice, this prior will be assumed independent of prior associations *D*_1:*i*−1_. The resulting hypothesis likelihood is then obtained by multiplying with the likelihood of the parent node as expressed in (9). We evaluate the spatial likelihood by taking a small set *N*_*d*_ of recently added local features that together are enough to determine a pose registration *including* the current feature *y*_*i*_ and data association *D*_*i*_. This pose registration is obtained by minimizing the squared error between the local and global map on that horizon:

$$L^{*}=\min\frac{1}{N_{d}}\overset{N_{d}}{\underset{k=1}{\sum }}w_{k}e_{k}^{2},$$ (11)

where the error term is either the Euclidean or the projected distance:

$$e_{k}^{2}=\left\{\begin{array}{c} \underset{s\in \left\{1,2\right\}}{\sum }∥n_{k}\cdot \left(m_{k}-\left(Ry_{k}^{s}+t\right)\right){∥}^{2},\;\;\;\text{for lines}\;\\ ∥\left(m_{k}-\left(Ry_{k}+t\right)\right){∥}^{2},\;\;\;\text{for points},\; \end{array}\right.$$ (12)

With *R* and *t* the rotation matrix and translation vector that together form the registration pose. The upper expression in (12) is used for matching line segments to lines using the normal *n*_*k*_ for both line endpoint $y_{k}^{1}$ and $y_{k}^{2}$ . The lower expression is used for matching regular point positions. The feature set used in (11) includes the current candidate feature, allowing us to evaluate the *lack-of-fit* with the new feature influencing the registration. The errors are illustrated in Figure 3. We treat corner points as points in this registration and disregard their orientation in obtaining the registration. The unnormalized likelihood is now determined by considering the error distance of the points, either Euclidean or projected, after aligning them using the obtained registration:

$$p\left(e\right)=\overset{N_{d}}{\underset{i=1}{\prod }}\frac{1}{\sqrt{2\pi \sigma_{i}^{2}}}\text{exp}\left\{-\frac{e_{i}^{2}}{2\pi \sigma_{i}}\right\}$$ (13)

$$\ln L\left(Y^{*};D_{i:1},M\right)\approx -\frac{1}{N_{d}}\overset{N_{d}}{\underset{i=1}{\sum }}\frac{e_{i}^{2}}{\sigma_{i}^{2}}$$ (14)

where *σ*_*i*_ is the error covariance of feature *i* on the local map.

### 4.4 Implementation

**FIGURE 5:**
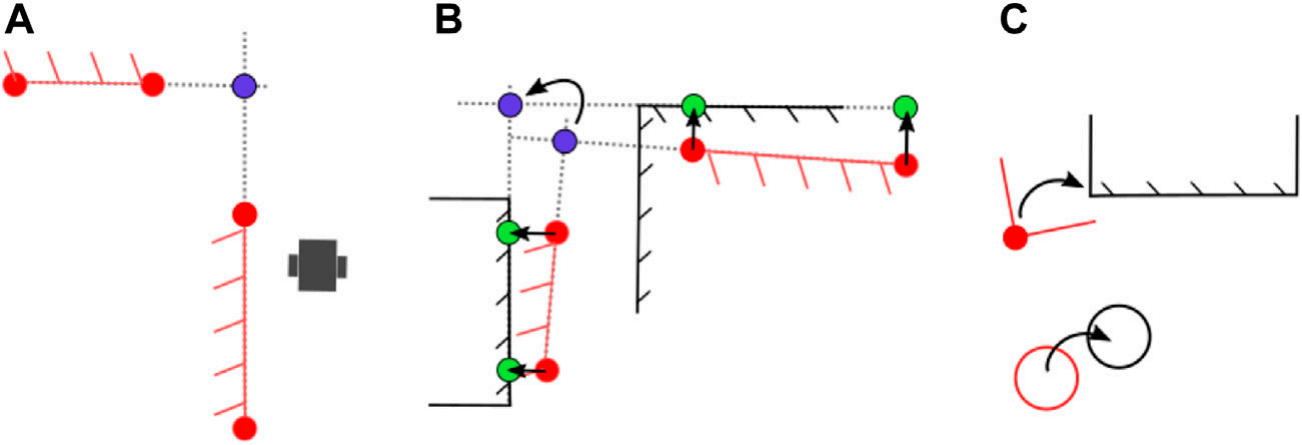
**(A)** Two (partial) line segments in the local map. **(B)** A pre-alignment step based on the virtual intersection point (blue) allows to acquire an initial estimate. We also use two point-correspondences or a single corner to obtain this pre-alignment, depending on which is most recently available. The segment endpoints are projected onto the global map line segments (green) to include them in the registration. The blue point is not used because it is only measured by extension. **(C)** Corners are treated as points in registration, similar to circles.

We use the well-known singular value decomposition (SVD) approach to obtain the solution to (11), since correspondences are already provided by the hypothesis under consideration. Figure 5 schematically shows how we use a pre-alignment step based on line intersection points to first determine an approximate registration. This allows to then project the line segment endpoints onto the infinite line extensions on the global map and incorporate them into the registration as points, resulting in an iterative solution. We employ a very coarse gating procedure for hypotheses based on the global pose to avoid pre-aligning and registering features unnecessarily.

For the weights in (12), a covariance estimate can be used from the local mapping. However, we supply simply unit weights because the covariance matrix obtained from the local map is, unlike the maximum a posteriori estimate, not necessarily an accurate estimate due to linearization and assumption of measurement independence. The method we propose should not be overly sensitive to these kind of modeling decisions. Some special attention has to be paid to the instances where the features do not permit a unique registration. In these cases, we evaluate a similar error metric for the *feature distance* that can be obtained without considering a registration. We distinguish between the following cases:

• The initial feature; in this case we only consider the descriptive prior likelihood for the data association.
• Parallel line segments; in this case we evaluate the distance between the segments.
• The *unmapped object* option; We use a heuristic probability that is small, but data driven estimates may provide better results that reflect sensor and environment characteristics.
• Any other case; where we can evaluate the likelihood as explained previously.

### 4.5 Pruning

**FIGURE 6:**
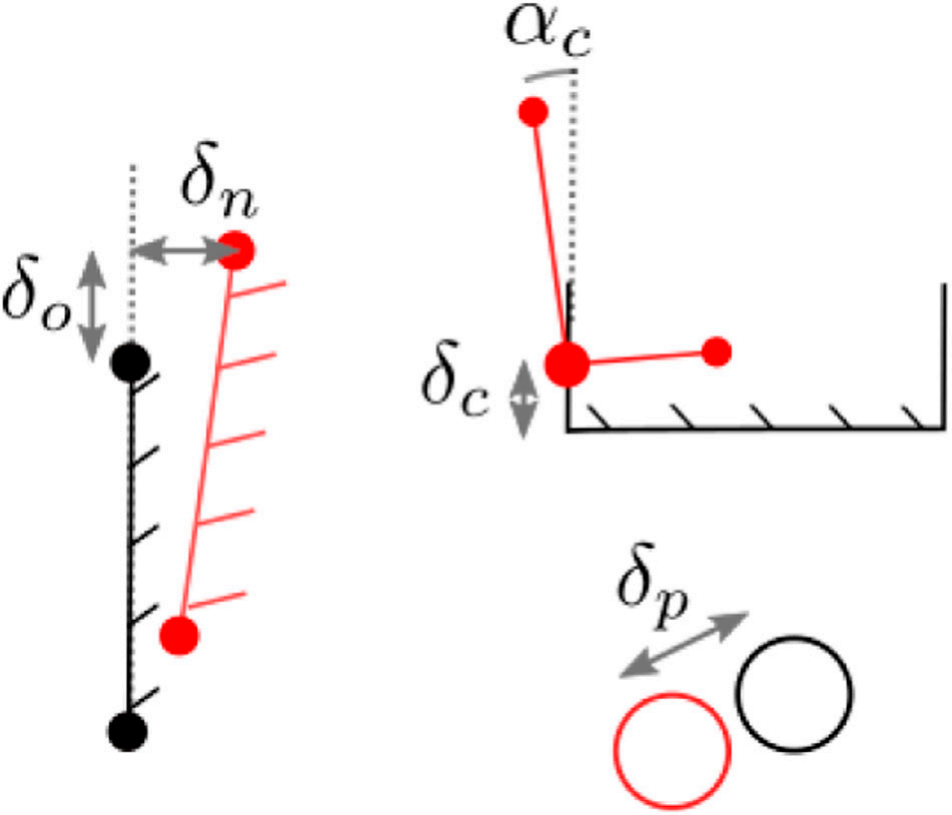
The thresholds that are checked to decide if a hypothesis should be rejected.

We will now introduce the mechanisms we employ to remove unlikely hypotheses from the tree. When considering a new local feature, first a gating step checks for every global map candidate whether the shape and size of the feature correspond and—if a pose estimate is available—whether the feature is roughly at the correct location. The latter gate is to prevent unnecessary evaluation during the spatial congruency check. For the congruency check, a small horizon of features including the new association candidate is registered according to criterium 12 and checked for errors that exceed a spatial threshold, as shown in Figure 6.

If any of the features exceeds this threshold the pairing hypothesis is rejected. Early pruning will limit the tree growth and prevent combinatorial explosion. However, it will not stop an existing hypothesis from expanding, even if there are no new local features supporting it. This is due to the explicit *not-on-map* option that is always expanded to allow delayed evidence^4^. To this end, we remove *redundant* hypotheses by checking whether hypotheses make similar associations in their history (excluding not-on-map) and keeping only the maximum likelihood hypothesis among them. Formally, for every leaf node (i.e., hypothesis), we recursively evaluate the parent nodes up to depth *N*_sim_ and consider leaf nodes similar if and only if they do not contain *-associations and all associations are the same. We then only keep the leaf node that has the highest likelihood. We also limit the number of consecutive not-on-map associations allowed for any hypothesis to γ_*_ to prevent unbounded tree growth. This parameter balances recovery potential with computational effort. Finally we have a pruning step that reduces the number of hypotheses to a fixed maximum *N*_max_, either based on likelihood or amount of *not-on-map* associations. The latter keeps *at least* *N*_*p*_ hypotheses, by choosing the smallest n_*_ where any hypothesis with more then n_*_ *not-on-map* associations is removed. This procedure is applied after convergence of the hypotheses to leave only a small set of *back-up* hypotheses remaining to recover locally from a false association.

Algorithm 2 The algorithm used to evaluate the hypotheses once a new set of local map features is available. It assumes that the tree already contains one or more levels. If the tree is empty, only feature shape is considered for the first local map feature.

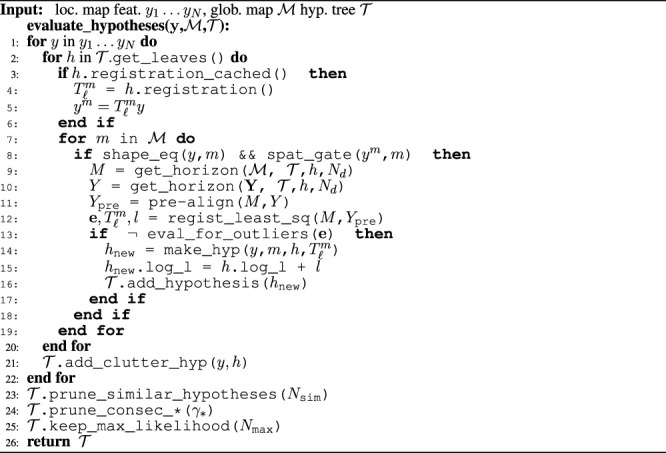

### 4.6 Implementation Considerations

In our work we do not explicitly represent probability distributions over a global pose. This sets our method apart from pose-based filtering methods. However, determining a maximum likelihood pose using our methods is straight forward given the data associations, and the registration obtained by (11) can be used for this purpose. A consequence is, furthermore, that we have access to a locally accurate, association-labelled map which may be used by motion planners. This is valuable, for instance, in a hallway where two parallel sides are annotated and permit a driving actions without relating to a pose. A consequence is that we need to determine the length of the local horizon we consider. The local map is chosen sufficiently large to enable considering a horizon of features for global association, but should not be seen as a means of closing *large loops*, i.e., loops that require a substantial drift to be corrected. Because this process is potentially error-prone and unnecessary for our localization objective.

Finally, if the objective under consideration changes to metrically accurate tracking, it may be favourable to replace our *breadth-first* expansion approach to *depth-first* tree expansion. In that case, one can provide the local graph with *global metric priors* obtained from the associations to obtain a more accurate estimate of the map position under the assumption that the map is of reasonable metric accuracy in the first place. backtracking in this depth-first approach based on feature monitoring to recover quickly from false associations is a topic for future work.

## 5 Experimental Evaluation

**FIGURE 7:**
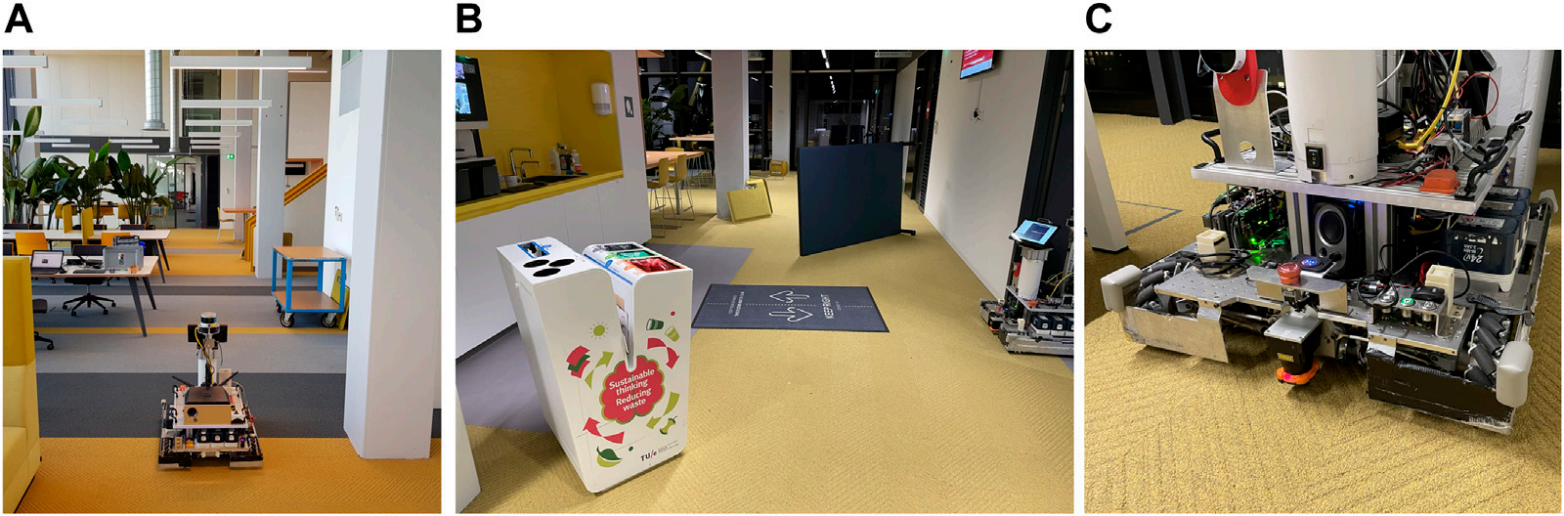
**(A)** The robot platform in the Atlas environment, the position is indicated in the map in Figure 9 position 1 (oriented towards the left). The situation shown is used as scenario A, where unmapped items are on the map in the form of chairs, tables and carts. **(B)** Some of the disturbances added to a part of the map to purposely obscure or mimick features for scenario **(C)** A close up of the robot platform with the planar LiDAR sensor.

In this section, we evaluate our approach in an indoor environment for which a map is available. We use this map to localize a teleoperated robot and we compare our method to the well-known AMCL (Fox, 2003) particle filter implementation. AMCL is openly available and uses a different paradigm for localization based on grid maps and beam-hit sensor models, which can be applied to global localization. We will validate and compare our method using criteria for success ratio, convergence speed and metric accuracy for different starting locations and different degrees of disturbance and prior knowledge. The real world experiments allow us to assess the performance qualitatively in scenarios with varying levels of uncertainty that we can add or remove in the form of prior knowledge and clutter. We use a custom-made platform equipped with mecanum wheels, wheel encoder odometry and a Hokuyo UTM30-LX 2D LiDAR scanner mounted upside down, close to the floor (Figure 7). Due to its mounting position, we have a 180° field of view consisting of 720 scan points. We choose this sensor type for evaluation because it is often encountered on mid-range existing robot platforms in real world applications.

### 5.1 Indoor Environment

**FIGURE 8:**
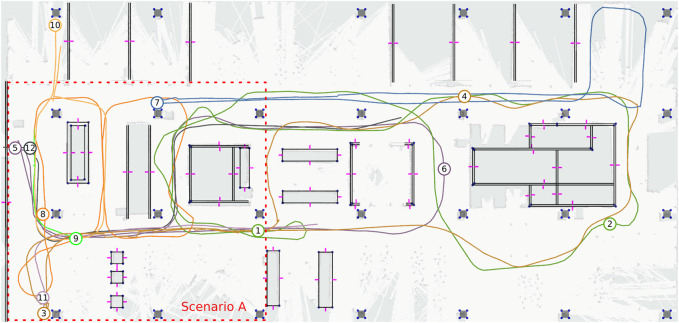
A map of the environment, with the trajectories driven by the robot indicated as paths and their starting locations (in different colors for clarity). The map contains line segments, corners (blue points) and visible normals indicated in purple. The world contains unmapped objects that are encountered during every day use of the environment. To show how these unmapped items influence the LiDAR readings, an occupancy grid map has been aligned to the map for visualization purposes only. The features shown are linked to semantic object instances, but these are omitted in the figure for clarity. The starting region for scenario A is shown in red.

We use a part of the Atlas building on the campus of the Eindhoven University of Technology for the experimental evaluation. Items that are moved often such as chairs, tables, carts and dustbins are not represented on the map that we use (sized approx. 20 × 41 m) and form the *unmapped object* that our method should be robust against. Furthermore, in part of our data set, bystanders disturb the view of the robot and we place objects in the scene that either obscure or resemble the static features that our method uses, which are likely scenarios. The map of the environment together with the initial starting positions, and a sub-imposed grid map is shown in Figure 8. Note that this is a SLAM-generated map that is only used for illustration. Based on the static vector geometry in Figure 8, we will generate a new grid map containing only the static parts used for our comparison. In Figure 7 we show the environment, some added disturbances for the final scenario and the robot platform.

### 5.2 Evaluated Scenarios

We consider the following scenarios labeled A-C:

1. This scenario considers two initial positions in the map, that are all within a starting region (sized approx. 15 × 15 m) known to the robot, shown in Figure 8.
2. This scenario considers ten different initial positions all over the map, where the starting region is unknown to the robot, containing only encountered disturbances in case of the real environment (including people).
3. This scenario considers added disturbances to the environment, in both simulation and real world, in the form of deliberately obscured features.

The scenarios are evaluated for a total of 12 different starting positions. The final scenario (C) is evaluated for two initial positions, accounting for a total of 14 evaluated global localization challenges. Our localization method exploits the initial knowledge in scenario (A) by transforming the starting position of the robot in the local map to the global map and rejecting hypotheses that were not started within the indicated region. For AMCL, the initial sample set is confined to the region.

### 5.3 Comparing With Monte-Carlo Localization

**FIGURE 9:**
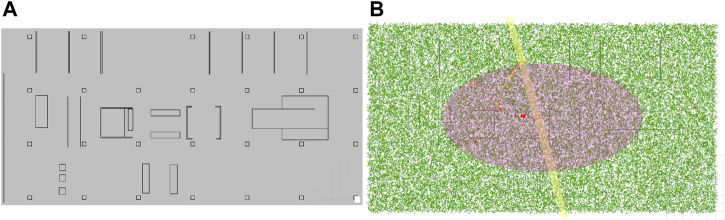
**(A)** The grid map containing the static geometry as generated for AMCL localization. **(B)** The initial particle spread in AMCL, consisting of 50,000 particles.

We compare the performance of our global localization approach with the monte carlo localization (MCL) approach based on grid maps. The reason is that in this localization paradigm, the robot has access to all geometry in the environment, due to the grid cell decomposition and pose sampling approach. In contrast to our proposed method, AMCL uses random (re-)sampling of hypotheses. We will run AMCL five times for every trajectory to account for this randomness (leading to a total of 70 AMCL runs). We choose the ROS AMCL implementation as it is a very commonly used software package using an adaptive sampling scheme. For this comparison, we generate the grid map for AMCL based on the vector representation of the static geometry in the environment. This vector map was made manually by referencing the actual environment and only includes objects on the map that do not move (i.e., no moveable furniture, tables, carts, trash bins). The grid map is shown in Figure 9 and was generated automatically from the vector representation in Figure 8, by setting grid cells to occupied if they cross the vector geometry. This way, both our approach and AMCL rely on a representation of the environment that can be obtained from a sensor-independent source that does not contain all the move-able geometry (*unmapped objects*) in the environment.

### 5.4 Configuration of the Approaches

In our feature-based approach, we select a minimal length of 0.2 m for corners and 1.5 m for line segments before taking them into account as features. The latter is heuristically chosen to avoid the detection of open doors as line segments. No circle shaped features are present in this environment, so they are not used. We trigger a perception update after every 0.05 m of driving and 0.05 radians of rotation (which in practice is higher because of loop time constraints, which we will consider later). We only trigger association tree evaluation when new features are available on the local map with at least three measurement from different poses. The tree evaluation parameters are given in Table 1. We show the grid map that was generated for AMCL in Figure 9, with a discretization of 0.05 m. For the AMCL configuration, the parameters that we used are in Table 2. We choose the likelihood field sensor model over the beam-based model because it showed better results by allowing to simulate more particles. We increase the initial particle count to 50,000, which is found to be the maximum reasonable amount of particles that our system is able to process. The particles are spread evenly over the map by uniform sampling.

**TABLE 1:** The configuration parameters used in our method for evaluating the hypothesis tree.

Param	Value	Descr
*N*_max_	200	max. # hypotheses
*N*_sim_	2	similar associations before prune
γ_*_	3	max. consecutive not-on-map assoc
*σ*_*e*_	1	error covariance
*p* (*)	0.1	probability not-on-map assoc
*δ*_*o*_, *δ*_*n*_, *δ*_*c*_, *δ*_*p*_	0.5	error tolerance before prune
*α*_*c*_	0.5	error angle tolerance before prune

**TABLE 2:** The configuration parameters used for running AMCL.

Param	Value
*N*_min_*particles*_	100
*N*_max_*particles*_	50,000
*N*_*kld*_*err*_	0.01
*N*_*update*_*min*_*d*_	0.10
*N*_*update*_*min*_*a*_	0.20
*N*_*resample*_*interval*_	2
*N*_*transform*_*tolerance*_	0.1
*N*_*recovery*_*alpha_slow*_	0.0
*N*_*recovery*_*alpha_fast*_	0.0
*N*_*laser*_*max_range*_	LiDAR value (30)
*N*_*laser*_*max_beams*_	30
*N*_*laser*_*z*_*hit*_	0.95
*N*_*laser*_*z*_*short*_	0.1
*N*_*laser*_*z*_ max_	0.05
*N*_*laser*_*z*_*rand*_	0.05
*N*_*laser*_*sigma*_*hit*_	0.2
*N*_*laser*_*lambda*_*short*_	0.1
*N*_*laser*_likelihood_max_dist_	2.0
*N*_*laser*_*model_type*_	likelihood_field
*N*_*odom*_*model_type*_	omni
*N*_*odom*_*alpha1*_	0.2
*N*_*odom*_*alpha2*_	0.2
*N*_*odom*_*alpha3*_	0.2
*N*_*odom*_*alpha4*_	0.2

### 5.5 Performance Metrics

We will run the algorithms real time on a laptop with an Intel Quadcore i7-7700HQ CPU @ 2.80 GHz × 8 processor. We evaluate the following performance metrics:

• Succes: We define success as the maximum likelihood hypothesis reaching and maintaining an error of less than 1.0 m within the 60 s time frame of the run.
• ML dist: We define the maximum-likelihood (ML) distance as the distance driven in which the previous succes criterium is met.
• Self-rep. distance: We define self-reported distance as the distance in which our method reports no hypothesis that exceeds the maximum deviation of 1.0 m from the weighted average of all hypotheses that permit a pose location. For AMCL, we use a threshold of 2 m on the x and y pose covariance.
• ML error: We report the error after the particles have converged to the right location, to indicate error in tracking mode.
• Max Hyp: We report the maximum number of hypotheses before and after the particles have converged to the right location.

For AMCL, the indicated values are the average over all successful runs for that initial positions. We will also show some selected results regarding semantic associations and CPU usage for our approach.

## 6 Results and Discussion

**FIGURE 10:**
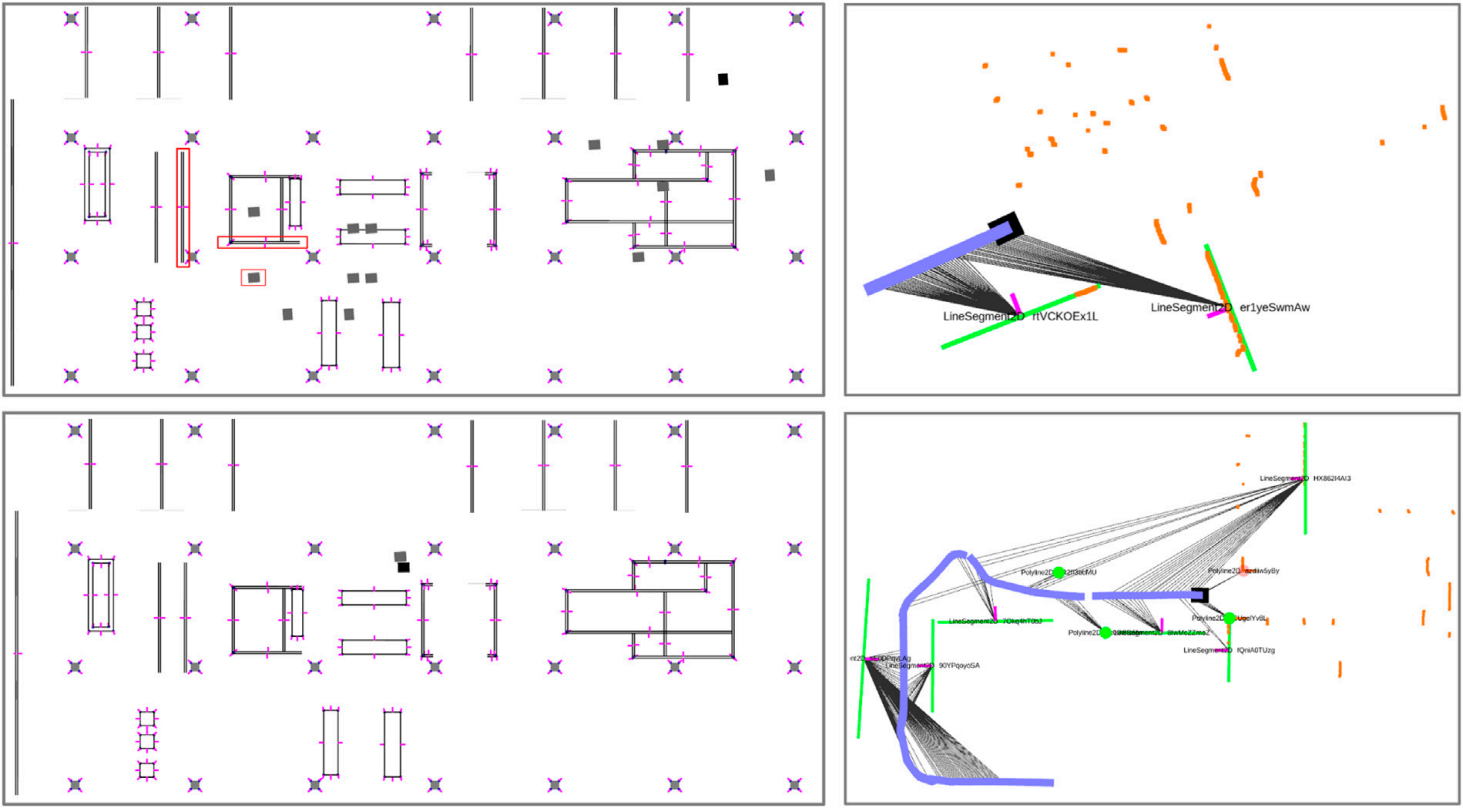
The top left map shows the global hypotheses that are created based on the local map at the top right. The correct hypothesis and feature pairings are indicated by red squares. The local map shows the LiDAR points in orange and the local map in green. The bottom figures show the global and local map after localization is achieved. The ML hpyothesis is show in black a number of *back-up* hypothesis in grey. One of them is significantly drifted because of a different assumption made recently. The others are covered by the ML hypothesis.

**FIGURE 11:**
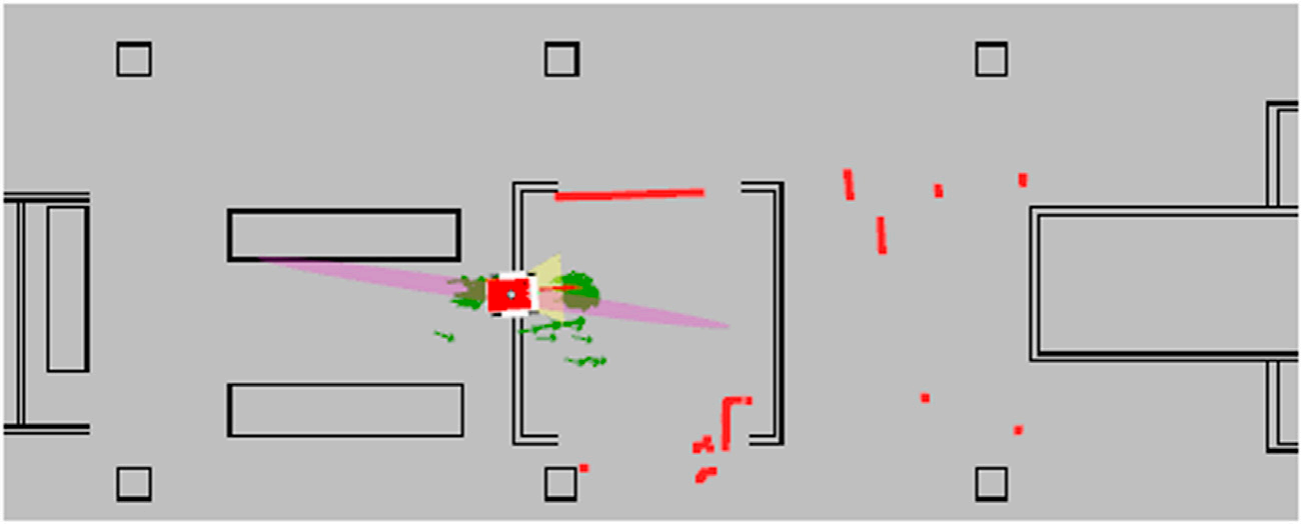
The AMCL method failing to converge, and providing a number of wrong hypotheses while including a maximum likelihood estimate that is incorrect and intersects with occupied geometry. The red square in the center is the robot position estimate and the green arrows are particles. The LiDAR points are shown in red as well.

The results of the localization are shown in Table 3. For the smaller initial region, our method achieves localization within 1.7 and 4.9 m for A1 and A3 with a maximum number of 200 hypotheses, which reduces to only resp. 8 and 7 hypotheses after self-reported localization. AMCL also achieves localization successfully when the initial pose is confined to the smaller region. When we increase the possible initial location to the entire map (scenario B1-B10), we see that our method still achieves localization in 9/10 runs, with distances ranging from only 1.1 m for B5 to 10.3 m for B10. The output of our method before and after localization is shown in Figure 10. The symmetries occurring from the feature associations are evident. The pattern shown here by two line segments with visible normals shown is already a very strong indicator of location. After localization, the figure shows the back-up hypotheses that enable to recover from wrong associations during tracking. Self-reported distances are usually significantly longer because alternative hypotheses still exist that offer alternative explanations of the local map. In some cases, such as B9, the self-reported distance is too low. This is due to only pose-enabling hypotheses being evaluated for convergence, and many hypotheses that do not permit a pose are present and appear to be correct in light of later evidence. For AMCL, we see that for some runs, such as B3, B8 and B9, successful localization is consistently achieved. In the case of B9, AMCL is very fast to converge to the right location. In B9, as is difficult to see in Figure 8, the robot rotates in place for a full round first, then moves to a second location and rotates again, exposing significant geometry of the environment without many disturbing elements present in the environment. Our method takes longer to localize in this scenario, because we rely on detecting stable features first from the geometry. In scenario B7 our method is not able to localize whereas AMCL is able to do so in 3/5 runs. Our method, in this scene, had trouble with the people and furniture in the open space to the left of the robot, which caused drift in our local map. In run B2, B4, B5 and B6, we see that AMCL is consistently not able to localize the robot. An example of this localization error is shown in Figure 11. Especially in the right half of the map (as can be seen in Figure 8), there is clutter present in the B-scenarios. While our method does localize, it results in erroneous hypotheses taking longer to be removed. Finally, in the C-level scenarios we see that AMCL is able to localize successfully (5/5 runs) in scenario C12 and sporadically in scenario C11. For our method, C12 is problematic because most of the objects mapped locally are actually clutter objects that have been placed to disturb the environment. This is shown in Figure 7B and in the left image of Figure 13. The middle and right image also show scenarios in which clutter appears in the local map that has to be dealt with (i.e, a dustbin and a open door appearing as a corner).

**TABLE 3:** Results of the localization using our method and AMCL. The predicate *succesfull* is *yes* when the robot localizes accurately with respect its ML hypothesis error stays localized for the remainder of the path. The distance needed to acquire localization is both expressed as actual localization of ML and as self-reported localization (by assessing convergence). Note that self-reported localization can be reported erroneously (i.e.,. false confidence). The average ML error after localization is also reported and the average number of hypotheses used both before and after self-reported localization has been achieved.

	Scenario	A		B										C	
	Descr	Init Region		Total map										Clutter	
	Path	A1	A3	B1	B2	B3	B4	B5	B6	B7	B8	B9	B10	C11	C12
ours	succesfull	yes	yes	yes	yes	yes	yes	yes	yes	no	yes	yes	yes	yes	no
	actual dist ML	1.7	4.9	1.7	8.3	4.9	5.3	1.1	7.2	n/a	5.5	7.1	10.3	16.4	n/a
	self-rep dist	13.2	4.9	16.3	22.0	8.4	24.1	15.7	20.9	16.9	5.5	4.0	6.0	2.9	9.1
	ML avg er aft con	0.05	0.33	0.07	0.23	0.28	0.36	0.13	0.25	n/a	0.24	0.51	0.16	0.08	n/a
	max # hyp bef con	200	200	200	200	200	200	200	200	200	200	200	200	200	200
	max # hyp aft con	7	9	7	10	7	5	33	11	n/a	8	7	5	6	n/a
AMCL	succesfull	5/5	5/5	2/5	0/5	5/5	0/5	0/5	0/5	3/5	4/5	4/5	3/5	2/5	5/5
	actual dist ML	5.2	6.2	3.9	n/a	9.3	n/a	n/a	n/a	6.5	10.3	1.2	6.3	13.1	8.0
	self-rep dist	5	6.9	6.4	n/a	13.3	n/a	n/a	n/a	8	12.2	2.9	7.4	12.9	8.7
	ML avg er aft con	0.19	0.32	0.18	n/a	0.29	n/a	n/a	n/a	0.2	0.17	0.25	0.36	0.42	0.22
	max # hyp bef con	5e4	5e4	5e4	n/a	5e4	n/a	n/a	n/a	5e4	5e4	5e4	5e4	5e4	5e4
	max # hyp aft con	5e4	44217	5e4	n/a	5e4	n/a	n/a	n/a	17378	28235	13527	5e4	5e4	5e4

### 6.1 Associations and Computing Time

**FIGURE 12:**
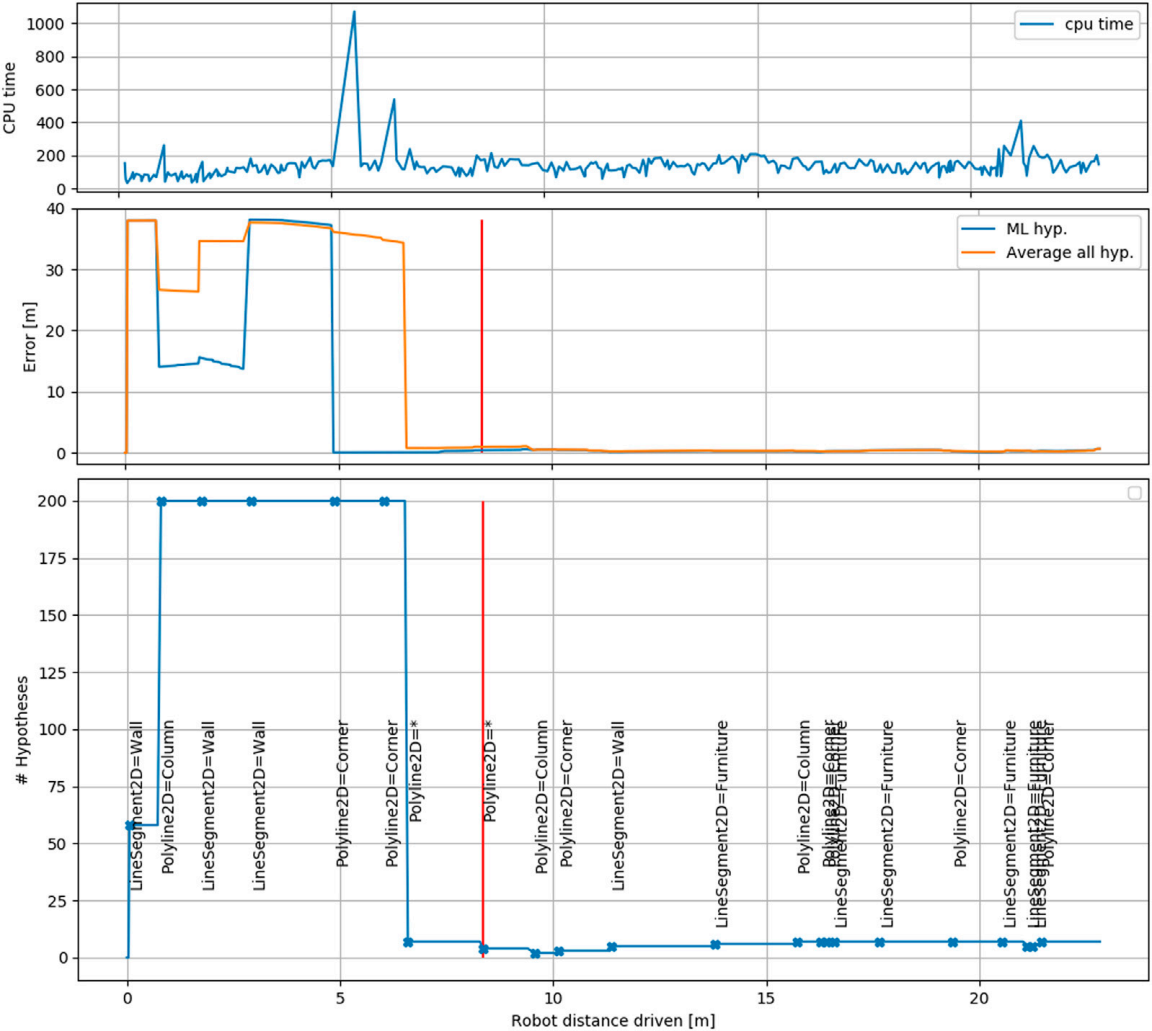
The CPU time, error and hypotheses shown for B3. The middle figure shows the error for both the maximum-likelihood hypotheses and the average over all hypotheses that permit a pose registration. The moment where our approach marks itself as localized is shown in red. Below, the number of hypotheses is shown, together with events that indicate that a new association is evaluated. The association for the ML hypothesis is shown in text (* indicates unmapped object). The top figure shows CPU cycle time (milliseconds) for session B3. Within a cycle, the local map is always updated and the associations are only updated if a new stable local feature is available. The latter causes the distinct peaks in processing time.

Figure 12 shows the number of hypotheses and error in time and illustrates an attractive property of our approach. We maintain a smaller number of hypotheses on the basis of symbolic associations, especially after convergence. Furthermore we make the associations very explicit. The new associations made by the ML hypothesis are indicated in the graph as events. The hypothesis tree is only evaluated at these instances for the new evidence. The resulting CPU cycle time is shown in Figure 12. The distinct peaks are caused by evaluation of the tree, whereas the local map update is usually between 100 and 200 ms. Note however that our current C++ implementation was written as a prototype with extendability in mind and has not been optimized for execution time. We expect that computation times of less than half the current times are obtainable.

**FIGURE 13:**
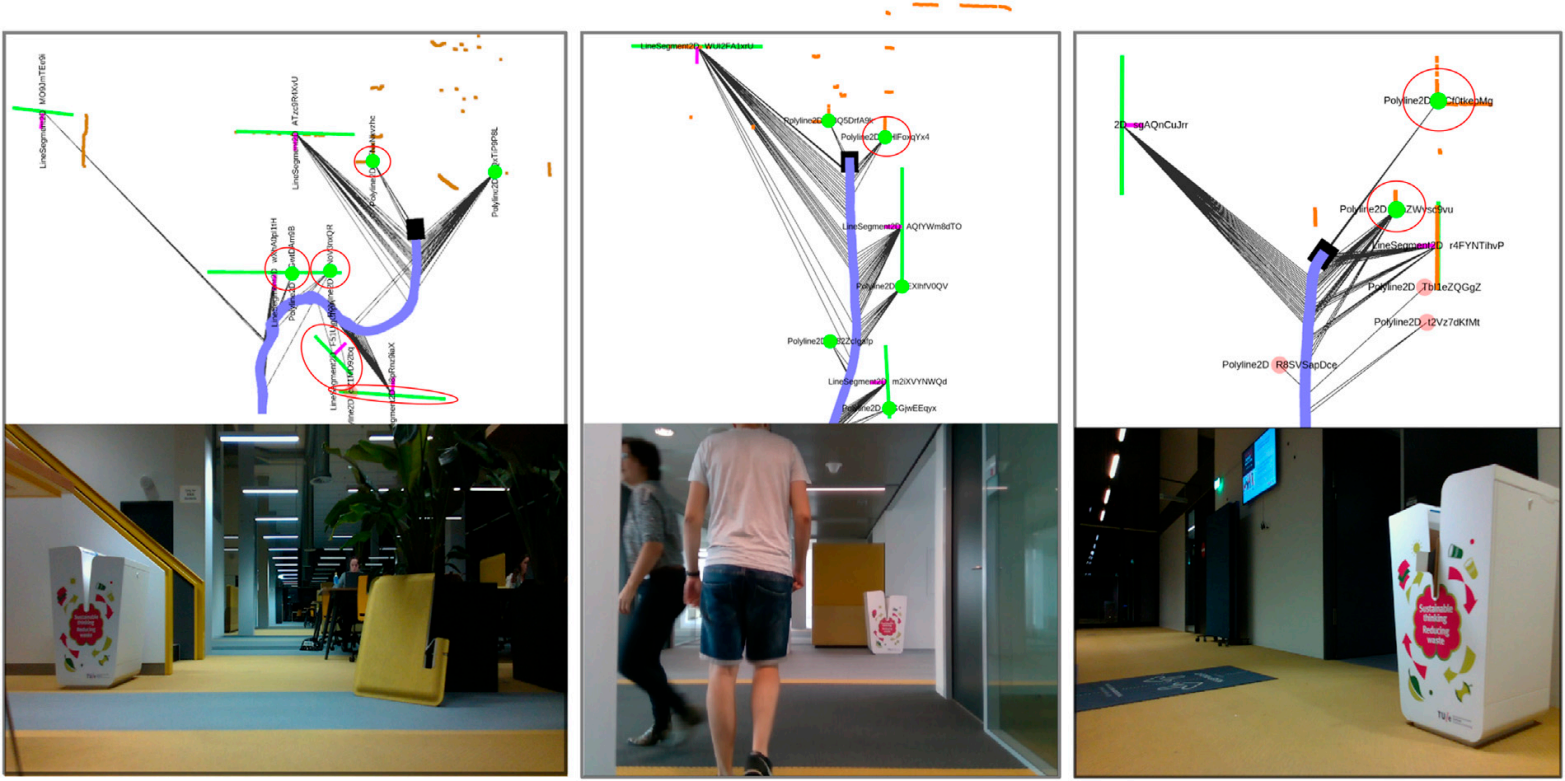
Three situations in which the local map contains objects that are not on the global map (marked by red circles). The camera images from the robot’s camera have been logged for verification purposes. The first situation is scenario C12 where disturbances have been added on purpose. The second scenario is B5 where the scene is disturbed by pedestrians walking in front of the robot. The third scenario is B10, where the robot leaves a room and spots a dustbin and an open door as corner features. Both are not on the global map and require sufficient not-on-map assumptions to survive the pruning process.

### 6.2 Limitations

The amount of hypotheses needed was determined by considering that we have 154 concave corners on our map. If the first detection would be a corner, we can evaluate all of them in light of the next pairing. This, however, shows a clear limitation in terms of hypotheses needed to cover larger maps in terms of feature count. One way to deal with this is to postpone evaluation and favor the evaluation of more unique features first (i.e., temporally out-of-sequence) such as large line segments, which we currently do not do. Furthermore, maintaining hypotheses on actual object-level or even spatial object patterns might significantly reduce the amount of hypotheses and computation time. Of course this computation time has to be balanced with the increased localization success that our method can achieve. Another drawback, in our experience, is the added complexity of our method over, e.g., Monte Carlo approaches. Our method requires bookkeeping of a local map, a feature detector and a hypothesis tree. Each step introduces possible failure points both inherent to their underlying assumptions and due to programming complexity. Furthermore, because we use features, we cannot localize when all features are obscured. For some environments, that are very cluttered this would be a likely scenario and describing the environment in terms of our feature shapes may not be feasible. Some of these drawbacks are handled more effectively by scan-based approaches that match features extracted specifically for the purpose of quick association retrieval from the same scanning device. However, these methods do not offer the same abstract notion of localization and cannot deal with existing geometry, suggesting possible complementary strengths in for example the area of keeping maps up to date. Our method provides the most opportunities in cases where prior existing maps are available and multi modal perception based semantic feature information has to be fused to localize a robot both effectively and in a semantically explainable way. This allows the robot to update which (semantic) features are salient and which should not be used in order to keep long term localization reliable and effective.

## 7 Conclusion

We showed a localization approach that uses a local feature map and a hypothesis tree to solve the localization problem in the association space. We compared our method with a grid based particle filter and showed that our method is able to localize in more cases, while in some cases the particle filter is more successful. The grid based particle filter does not represent hypotheses on a feature association level and requires a large amount of particles in order to localize successfully in larger environments. The results are dependent on the initial sampled distribution, which causes varying results in more difficult cases. Because the environment contains unmapped clutter, convergence can not be guaranteed. In theory, increasing the initial particle count could improve results, however with the current 50,000 particles we already run into the limits of processing capabilities. On the other hand, the method that we propose, association-based localization, does not rely on such an initial sampling and performs better in some of the scenarios that we investigated, if the amount of clutter is again limited. A benefit of our method is a significant decrease in the amount of hypotheses necessary to represent the robot’s belief about its location. And while our hypotheses are more expensive to maintain individually than the particles in a particle filter, the conceptual advantage of needing much fewer of them is promising. We foresee benefits when we consider the interpretation of the hypotheses by other parts of the motion stack (e.g. active localization planners) and the insight that our approach generates into the assumptions underlying the localization effort.

## 8 Future Work

Our work aims to provide robots with semantically explainable autonomy in environments for which prior maps are available. The next step is to include the robot’s actions into a task-based framework for fully autonomous navigation and recovery. We see two possible directions for extending the current work: 1) Pre process the map and use higher-level salient feature combinations (e.g. on object level) to improve performance and robustness and scale up to larger environments. 2) Use the association space of hypotheses in an explicit action selection policy. The first direction considers exploiting semantic and topological relations on the local map first, and creating indices into the global map to efficiently search for feasible locations based on these relations. These relations can also incorporate free space (e.g. a single column surrounded by a large area of free space) as this provides a very strong indication of feature location when seen in the LiDAR sensor. The second point is complementary, in the sense that actions of the robot can help to first gather this feature context (active sensing) before deciding to expand the hypothesis tree. In conclusion, our work provides a step in the right direction towards recoverable and semantically insightful robot navigation that can exploit information from multiple sensors locally first.

### A Local map Building

#### A.1 Measurements and Factors

**FIGURE 14:**
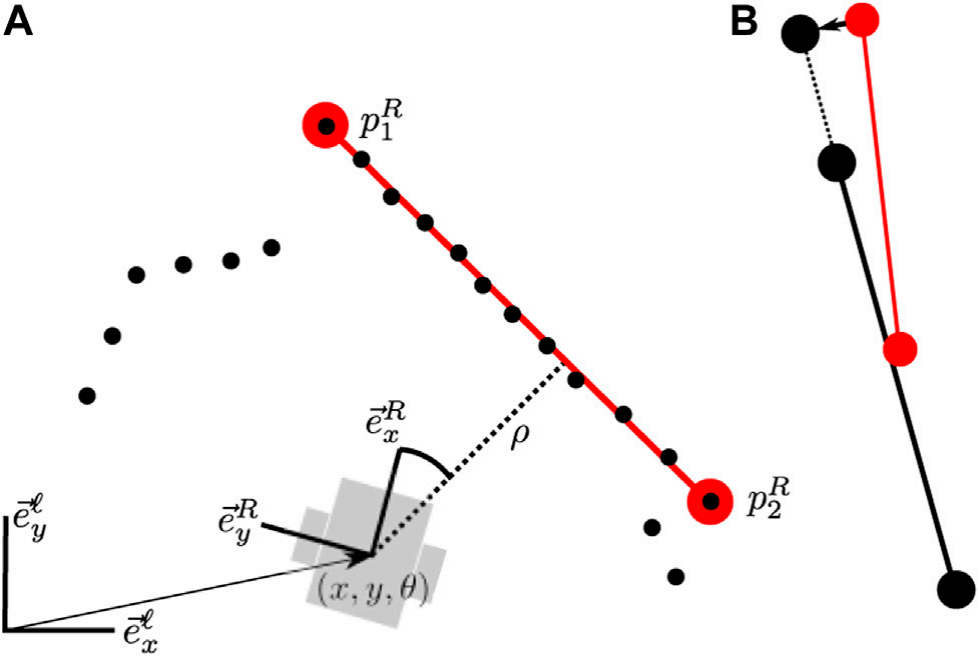
**(A)** The robot measures a sensor feature locally in the form of a line segment. The line segment gets added to the local map and a range-bearing measurement is added as well. **(B)** When a new sensor feature is associated with the same line segment on the local map, it is merged to form updated end points. The new range bearing measurement is also added between the robot pose and the line (but not shown).

**FIGURE 15:**
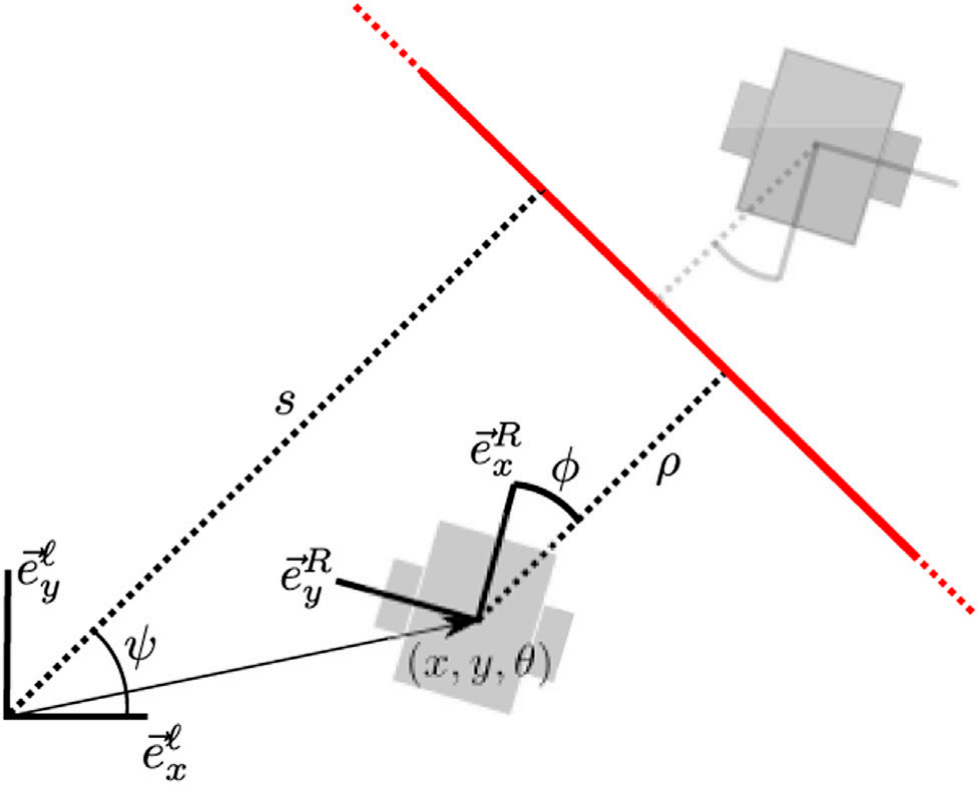
When the line segment is updated in the state estimate optimization, first a temporary range-bearing parametrization is generated in the local map as well to simplify calculation of measurement error and error Jacobians.

In this appendix we provide a more detailed explanation of how line measurements are used locally in our approach. To extract lines we use the implementation found in Gallant (2014); Pfister et al. (2003). A line sensor feature is represented by the point-pair coordinates (*p*_start_, *p*_end_) with respect to the robot and is extracted from a LiDAR scan (Figure 14). This line gets added to the local map (in frame *ℓ*) as a point-pair.A second partial line segment sensor feature can be associated with the same local feature. This association is made when both points of the new sensor feature fall within a distance *d*_*n*1_ from the infinite line extending from the line on the local map and one of the points is within the segment. To solve for the most likely position of the line in the local map given multiple measurements, the point-pair representation is temporarily converted to an infinite line with range-bearing parametrization (Figure 15).In order to optimize over both the robot pose and the line parameters, we create a factor for the measurement error, which is the difference between expected and measured (*ρ*, *ϕ*), given by:

$$e_{\rho }=\left\{\begin{array}{c} \rho -s+x\cos\psi +y\sin\psi \;\\ \rho +s-x\cos\psi -y\sin\psi \; \end{array}\right.$$ (15)

$$e_{\phi }=\left\{\begin{array}{c} \phi -\psi +\theta \;\\ \phi -\psi +\theta +\pi ,\; \end{array}\right.$$ (16)

where the latter case needs to be used when the robot is in the halfspace of the line that does not contain the local frame origin. To optimize over the robot pose and line parameters, we obtain the following jacobians:

$$\frac{\partial e}{\partial x}=\left\{\begin{array}{c} \left[\begin{array}{ccc} 0 & 0 & 1\\ \cos\psi & \sin\psi & 0 \end{array}\right]\;\\ \left[\begin{array}{ccc} 0 & 0 & 1\\ -\cos\psi & -\sin\psi & 0 \end{array}\right]\; \end{array}\right.$$ (17)

$$\frac{\partial e}{\partial y}=\left\{\begin{array}{c} \left[\begin{array}{cc} -1 & 0\\ -x\sin\psi +y\cos\psi & -1 \end{array}\right]\;\\ \left[\begin{array}{cc} -1 & 0\\ x\sin\psi -y\cos\psi & 1 \end{array}\right]\; \end{array}\right.,$$ (18)

again depending in which halfspace the robot is located. The implementation in GTSAM requires a custom implementation of the 2D line type with manifold traits and a custom factor. The type needs to have the interfaces retract () and localCoordinates () implemented to move between the group element and local coordinates. The retract () interface adds an increment in local coordinates to the line, and contains logic to always return a parametrization with a positive range *ρ*. The line factor introduced here, together with range-bearing factors for point measurements and relative pose factors for the odometry form are used to form a factor graph which is locally consistent around the robot. The result can be seen in Figure 2, where a number of lines, corners and circles have been measured multiple times and given unique id’s on the local map. The measurements taken from a certain robot pose are shown as grey lines.

**FIGURE 16:**
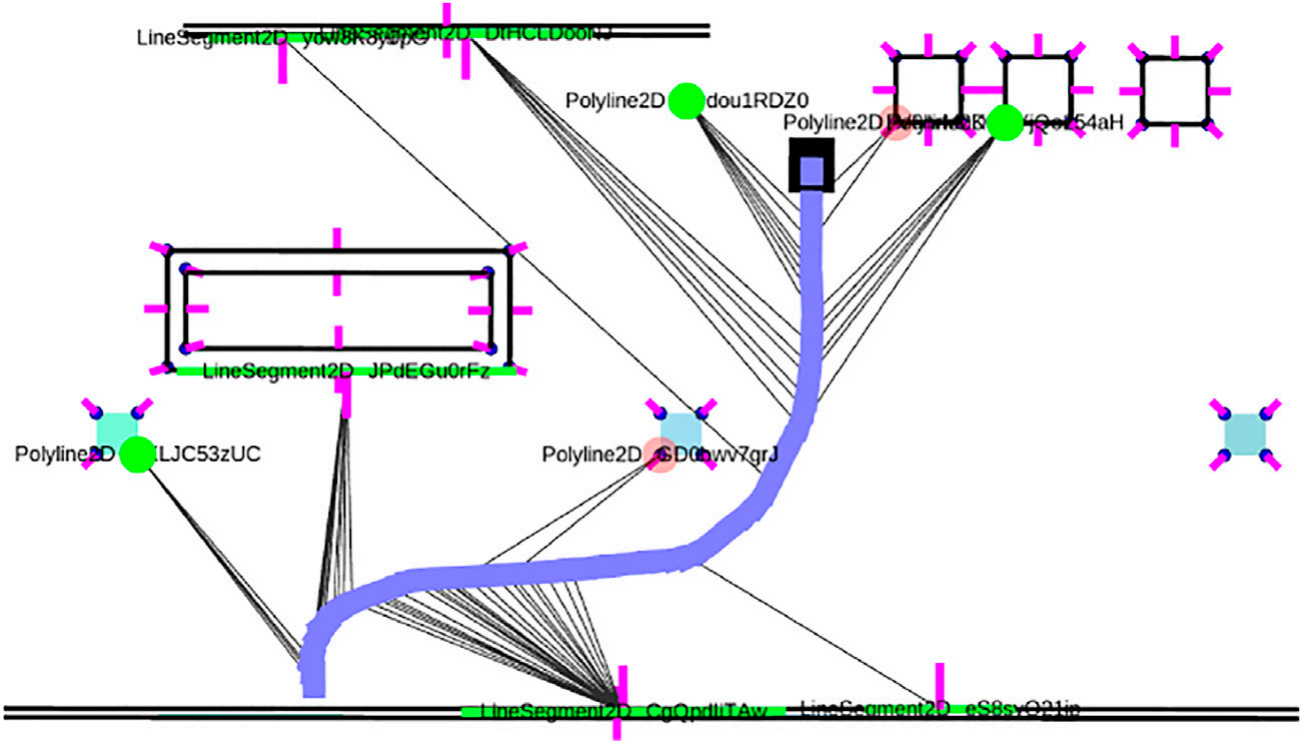
The local map of features (green) aligned against the global map, which serves as a prior using nearest neighbor data associations. The resulting robot trajectory is the MAP estimate of the robot poses in the global map. We used this method to obtain the ground truth estimates for our trajectories to verify our hypothesis approach. The robot trajectory is indicated by the blue line and the green and red circles and lines indicate features.

#### A.2 Improving Odometry With Local ICP

To improve local odometry in the presence of drift and time synchronization inaccuracies, a local *Iterative Closest Point* matching is used to obtain improved odometry by using two consecutive laser scans. The rationale behind this is that the *true odometry* is the one matching two scans perfectly, which is slightly different from the encoder odometry due to drift, timing mismatch and mounting position error. It must be noted, however, that ICP in environments where the static assumption is violated may possibly yield worse results than exclusively using odometry.

### B Tracking Mode

In this section we explain the tracking mode that was used to obtain the ground truth trajectories against the accurate map of the building. In tracking mode, the local graph as explained in the previous section is related to the global frame by making nearest-neighbor data associations with the global map, after an initial pose estimate has been provided by the user. These associations are then incorporated in the graph as location priors for the local features expressed in the global map, resulting in robot poses that are also valid with respect to the global map. This is visualized in Figure 16. The tracking mode can be started in our implementation by simply providing an initial pose estimate in RVIZ. For our ground truth, we manually supplied the starting position of the robot for each trajectory and verified the data associations that were made. The resulting robot poses at sample intervals were then used in Table 3 as ground truth.

## Data Availability

The raw data supporting the conclusions of this article will be made available by the authors, without undue reservation.

## Footnotes

1
https://gitlab.tue.nl/s135700/semantic_graph_localization

2 While a measurement set is technically a redundant notion, since measurements are already associated with a single feature, it makes bookkeeping and implementation more clear.

3 Except for the root hypothesis, which only serves as a placeholder for the first layer of children, and may hold optional prior information supplied by the user regarding location.

4 Note that we never refrain from making a *not-on-map* option, because erroneous pairings can appear confident due to error. The delayed-evidence mechanism is the major reason for robustness against this.
